# Association between various types of corticosteroids and mortality for severe community-acquired pneumonia in adults: a systematic review and network meta-analysis

**DOI:** 10.3389/fphar.2024.1479804

**Published:** 2024-11-26

**Authors:** Peng Wang, Jun Wan, Qiaoyu You, Yuxin Zheng, Wenhao Xu, Jialing He, Liyuan Peng, Cuyubamba Dominguez Jorge Luis, Yuning Feng, Ping Xu, Xinrong Li, Weelic Chong, Yang Hai, Lu Jia, Fang Fang, Yu Zhang

**Affiliations:** ^1^ Center for Evidence-Based Medicine, Affiliated Hospital of Chengdu University, Chengdu, Sichuan, China; ^2^ Department of Neurosurgery, West China Hospital, Sichuan University, Chengdu, Sichuan, China; ^3^ Clinical Medical College and Affiliated Hospital of Chengdu University, Chengdu University, Chengdu, Sichuan, China; ^4^ Department of Critical Care Medicine, Affiliated Hospital of Chengdu University, Chengdu, Sichuan, China; ^5^ Institutes for Systems Genetics, West China Hospital, Sichuan University, Chengdu, China; ^6^ Sichuan University Library, Sichuan University, Chengdu, Sichuan, China; ^7^ Tangshan Research Institute, Southwest Jiaotong University, Tangshan, Hebei, China; ^8^ Department of Medical Oncology, Thomas Jefferson University, Philadelphia, PA, United States; ^9^ Sidney Kimmel Medical College, Thomas Jefferson University, Philadelphia, PA, United States; ^10^ Department of Surgical Intensive care medicine, Shanxi Provincial People’s Hospital, Taiyuan, Shanxi, China

**Keywords:** corticosteroids, community-acquired pneumonia, mortality, network meta-analysis, SCAP

## Abstract

**Objective:**

This study aims to conduct a systematic review and network meta-analysis to evaluate the efficacy and safety of specific corticosteroids, including but not limited to hydrocortisone, methylprednisolone, prednisolone, and dexamethasone, in the treatment of severe community-acquired pneumonia (SCAP). Efficacy will be assessed using specific outcomes, such as 30-day mortality and the requirement for mechanical ventilation. Safety evaluations will include adverse events like gastrointestinal bleeding and healthcare-associated infections. The study seeks to address the gaps identified in the latest guidelines regarding the optimal use of different corticosteroid types and to provide recommendations for clinical practice.

**Data Sources:**

This study conducted a comprehensive search of Medline, Embase, and the Cochrane Central Register of Controlled Trials, covering the period from inception until 22 June 2023. Randomized clinical trials for corticosteroid use among adults with SCAP were collected.

**Study Selection:**

Two researchers independently assessed study eligibility based on titles and abstracts, with any disagreements resolved through discussion or consultation with a third researcher.

**Data Extraction:**

Two researchers independently collected and clarified study details, with a third researcher adjudicating in case of disputes.

**Data Synthesis:**

The data from 13 randomized clinical trials involving 2,495 patients, were analyzed using a random-effects model. Eleven trials were assessed as low risk, while two were assessed as high risk. Trials were rated as having low bias risk. Results, presented as risk ratios (RR) with a 95% confidence interval (CI), indicated that hydrocortisone outperformed prednisolone (RR 0.35; 95% CI 0.10–0.94), methylprednisolone (RR 0.41; 95% CI 0.15–0.89), and placebo (RR 0.35; 95% CI 0.16–0.59) in reducing 30-day mortality. A rankogram plot suggested that hydrocortisone had the highest probability of being the most effective treatment for this outcome within the analyzed group.

**Conclusion:**

In this network meta-analysis, while hydrocortisone showed greater efficacy than prednisolone, methylprednisolone, and placebo in reducing 30-day mortality in patients with SCAP, further Randomized Controlled Trials (RCTs) are required to confirm these findings before drawing definitive conclusions.

**Systematic Review Registration:**

https://www.crd.york.ac.uk/PROSPERO/display_record.php?RecordID=438389, identifier CRD42023438389.

## Highlights

• Question: What is the efficacy and safety of different corticosteroid treatments, in terms of type, for SCAP?
• Findings: In a comprehensive network meta-analysis of 13 randomized clinical trials involving 2,495 patients, hydrocortisone demonstrated superior efficacy compared to prednisolone, methylprednisolone, and placebo in reducing 30-day mortality.
• Meanings: Hydrocortisone emerges as a more effective option for reducing mortality in SCAP patients compared to other evaluated corticosteroids, providing valuable insights for clinical decision-making.

## Introduction

SCAP is one of the most prevalent serious infectious diseases globally, characterized by high morbidity and a mortality rate reaching 10%–20% (Nair and Niederman, 2021). Despite advancements in antibiotic therapies, the high mortality associated with SCAP remains a significant public health concern (Deshpande et al., 2020). Therefore, exploring more effective adjunctive treatment strategies is particularly crucial.

Currently, the latest guidelines recommend glucocorticoids as adjunctive therapy for SCAP, but there is still debate and a research gap regarding the optimal type to use (Martin-Loeches et al., 2023). Different glucocorticoids—such as hydrocortisone, methylprednisolone, and prednisolone—exhibit significant differences in mechanisms of action and immunomodulatory properties; even at equivalent doses, their clinical effects may vary (Demoly and Chung, 1998). For example, the CAPE COD trial (Dequin et al., 2023) demonstrated that hydrocortisone significantly reduced 28-day mortality, whereas another trial (Meduri et al., 2022) conducted across 42 Veterans Affairs medical centers found that methylprednisolone did not produce the same effect. This discrepancy in efficacy heightens uncertainty in clinical practice, preventing current guidelines from reaching a consensus on the optimal glucocorticoid type.

Therefore, systematically evaluating the efficacy and safety of different glucocorticoid types in SCAP patients is of paramount importance for optimizing treatment strategies. This study employs a network meta-analysis (Caldwell et al., 2005; Bafeta et al., 2014) to comprehensively compare the efficacy and safety profiles of various glucocorticoids in SCAP, aiming to fill this research gap and provide reliable evidence-based guidance for clinical practice.

## Methods

### Protocol and guidance

Network meta-analysis is a statistical method that allows for the simultaneous comparison of multiple treatment interventions. It integrates techniques from traditional meta-analysis by assessing three or more interventions through both direct and indirect comparisons. This approach enables researchers to evaluate the relative efficacy of various treatments within a single model, even in the absence of direct comparison data between them (Jansen et al., 2011). This study was conducted in accordance with the Preferred Reporting Items for Systematic Reviews and Meta-Analyses (PRISMA) guidelines (Hutton et al., 2015). The study protocol was registered with PROSPERO (CRD42023438389).

### Eligibility criteria

The eligibility criteria for this study were determined based on the PICOS Criteria (participants, interventions, comparators, outcomes, and study design). All included studies received ethical committee approval and obtained informed consent from patients or their legal representatives, where applicable. We included published RCTsthat met the following criteria.

1) Participants: The study population comprised adults (aged ≥ 18) who were diagnosed with SCAP, which was defined as community-acquired pneumonia accompanied by requiring ICU admission, or meeting either the criteria for severe pneumonia by the American Thoracic Society/Infectious Diseases Society of America (ATS/IDSA) (Mandell et al., 2007) or classified as risk class IV or V of the Pneumonia Severity Index.
2) Interventions: Systemic corticosteroid treatment, including but not limited to hydrocortisone, methylprednisolone, prednisolone, and dexamethasone. Corticosteroids could have been administered at any mode and for any duration in the management of pneumonia.
3) Comparison intervention: corticosteroid, standard therapy or placebo (when all groups received other agents, such as antibiotics).
4) Outcome: the primary outcome is 30-day mortality. In cases where actual 30-day mortality rates were not reported, we extracted mortality rates closest to 30 days, the secondary outcome was the need for mechanical ventilation and the secondary safety outcomes included gastrointestinal bleeding and healthcare-associated infections.
5) Study design: RCTs.

We excluded studies that exclusively included individuals with COVID-19 or HIV due to the distinct pathophysiological mechanisms, treatment protocols, and immune responses associated with these conditions, which may confound the interpretation of corticosteroid efficacy in SCAP. Additionally, studies involving topical or inhaled corticosteroids were excluded as they do not provide the systemic effects required for evaluating the impact of corticosteroid therapy on mortality in severe pneumonia cases. Information sources and search strategy.

A medical librarian (PX) developed search strategies in Medline, Embase, the Cochrane Central Register of Controlled Trials, and the World Health Organization International Clinical Trials Registry Platform from their inception until 22 June 2023. To minimize the risk of omitting important literature, we conducted manual searches of key journals and conference proceedings related to the field of community-acquired pneumonia and corticosteroid therapy. We scrutinized the reference lists of relevant articles to identify any additional studies not captured through the database searches. Furthermore, no restrictions were placed on language or publication status, allowing us to capture a broad range of studies from various sources. The detailed search strategy is outlined in Supplement S1. The terms “corticosteroids” “pneumonia” and “randomized controlled trial” were searched both individually and in combination. More information regarding the conducted search strategy can be found in the Supplement Table S1.

### Study selection

Two researchers (PW and FF) independently assessed the eligibility of studies by evaluating titles and abstracts retrieved from the electronic search. The full texts of the remaining relevant articles were also independently assessed for inclusion by these two researchers. In the event of any disagreements, a resolution was achieved through discussion or consultation with a third researcher (YZ).

### Data extraction

Two researchers (PW and WX) independently utilized a standardized data extraction form (detailed in Supplementary Material S2) to systematically collect key information from each study. Additionally, we assessed patient adherence in the included RCTs and found it to be good information included study population characteristics, types of corticosteroids used, control group settings, and primary and secondary outcome measures. To ensure accuracy and completeness, both researchers compared their extracted data, and any discrepancies were resolved through discussion or by consulting a third researcher (YZ). Additionally, we employed Covidence (www.covidence.org) (Harrison et al., 2020) software to manage the literature screening and data extraction process, which enhanced efficiency and minimized the potential for human error.

### Assessment of risk of bias and certainty of the evidence

In this network meta-analysis, we used the Risk of Bias tool to assess the risk of bias (Puljak et al., 2024) (www.riskofbias.info) in the included studies. This tool covers five key domains to evaluate different types of bias, including bias arising from the randomization process (D1), bias due to deviations from intended intervention (D2), bias due to missing outcome data (D3), bias in measurement of the outcome (D4), and bias in selection of the reported result (D5). Each domain assesses the following aspects:

D1: Bias arising from the randomization process: Evaluates the generation of random sequences and the balance between groups to ensure no selection bias.

D2: Bias due to deviations from intended intervention: Assesses whether the intervention was implemented according to the study design, with particular attention to allocation execution to avoid performance bias.

D3: Bias due to missing outcome data: Reviews the completeness of the outcome data and how missing data were handled to ensure that missing data do not impact the reliability of the results.

D4: Bias in measurement of the outcome: Analyzes whether the outcome measurement was subject to bias, ensuring proper blinding during the assessment to avoid detection bias.

D5: Bias in selection of the reported result: Evaluates whether there was selective reporting of certain outcomes, ensuring that all predefined outcomes were fully reported to avoid reporting bias.

Two reviewers (PW and WX) independently evaluated the risk of bias in the included trials using the Risk of Bias tool (Sterne et al., 2019), which comprises five domains. For each domain, each trial was assigned a study-level score indicating the level of bias risk, categorized as low, high, or some concerns. We assessed the certainty of evidence using the Grading of Recommendations, Assessment, Development, and Evaluation (GRADE) approach for our network meta-analysis (Brignardello-Petersen et al., 2018; Phillippo et al., 2019). Any differences in assessment were resolved through consensus, with involvement from a third author (YZ) to make the final judgment in cases where consensus could not be reached.

### Data synthesis

This network meta-analysis was conducted using R software (version 4.0.3) (www.r-project.org), (Shim et al., 2019) netmeta, and the gemtc package (version 1.0–1) (Neupane et al., 2014), which are based on the Bayesian framework and frequentist approaches. The purpose of this analysis was to compare multiple treatments using Markov chain Monte Carlo operation with vague priors (van Valkenhoef et al., 2012).

We employed a random effects model in our analysis to account for the expected variability among the included studies. Given the differences in study designs, patient demographics, intervention protocols, and outcome measures, a random effects model offers a more accurate estimation by accommodating variability in effect sizes across studies. This method contrasts with a fixed effects model, which presumes a uniform effect size and thus overlooks between-study heterogeneity. The random effects model was implemented using the gemtc package, which facilitates flexible modeling of heterogeneity among studies. To address heterogeneity, we incorporated study-level covariates where appropriate and conducted sensitivity analyses to evaluate the robustness of our findings.

For dichotomous variables, we estimated treatment effects using RR with a 95% CI (O'Rourke and Altman, 2005). Additionally, we ranked the interventions based on the primary outcome using a Rankogram plot. This plot shows the probability of interventions being ranked first, second, third, and so on.

### Subgroup analysis

We converted all steroid dosages to equivalent dosages of dexamethasone using the following conversion standards. Hydrocortisone 20 mg = Dexamethasone 0.75 mg; Methylprednisolone 4 mg = Dexamethasone 0.75 mg; Prednisolone 5 mg = Dexamethasone 0.75 mg; We performed the subgroup analysis based on the cumulative dexamethasone doses: lower cumulative doses of corticosteroids (total dose of ≤60 mg dexamethasone equivalent) and higher cumulative doses of corticosteroids (total dose of >60 mg dexamethasone equivalent). This categorization is based on previous studies, such as the one by Burçin Halaçli et al. (2023).

### Sensitivity analyses

We conducted four sensitivity analyses to explore the impact of certain factors on our analysis. The first sensitivity analysis excluded studies that only administered a single dose of corticosteroids. This was done because current pharmacological findings and guidelines suggest that prolonged corticosteroid treatment is necessary for severe pneumonia (Meduri et al., 2020). The second sensitivity analysis excluded studies that had less severe disease conditions. As there are variations in how SCAP is defined, we used the mortality rate of the control group as an indicator of disease severity (Fang et al., 2019). Therefore, we excluded the studies in the lowest quartile based on control group mortality rate. This helped us generate more robust and specific findings for severe cases. The third sensitivity analysis excluded studies that included patients with C-reactive protein levels below 15 mg/dL to verify the robustness of the research results under different C-reactive protein levels. The fourth sensitivity analysis was conducted by exclusively focusing on RCTswith a low risk of bias.

## Results

### Study selection and characteristics of included studies

**FIGURE 1:**
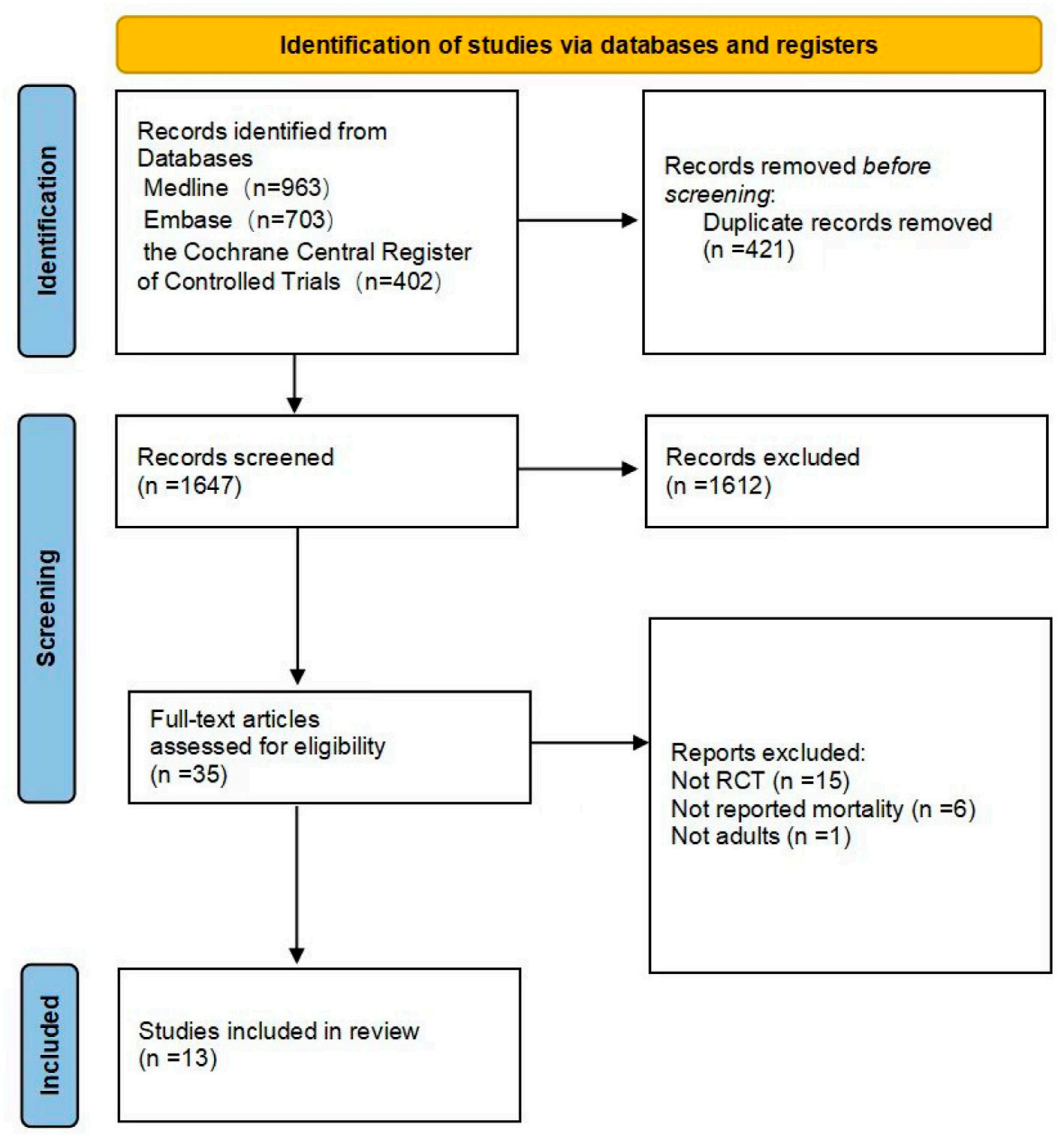
Search strategy and final included and excluded studies.

The study selection process is depicted in Figure 1. Out of the initial 2068 results, a total of 13 clinical trials comprising 2,495 patients met the eligibility criteria.

Table 1 presents the key characteristics of the selected studies. These studies were published between 1993 and 2023, with sample sizes ranging from 30 to 795 patients. Among the trials, 6 investigated the use of hydrocortisone, 4 explored methylprednisolone, 2 examined prednisolone, and 1 focused on dexamethasone.

**TABLE 1:** Characteristics of included studies.

Study	Patients, n	Male, (%)	Age, years	Disease severity		ICU admission, no. (%)		Mortality rate, no. (%)		CRP (mg/dL)	Regimen of corticosteroid	Cumulative equivalence dose (dexamethasone)
				CS	PC	CS	PC	CS	PC			
Dequin et al. (2023)	795	69.4	67	SOFA: 4 (3–6) PSI V: 181 (46)	SOFA: 4 (3–6) PSI V: 193 (49)	400 (100)	395 (100)	25 (6)	47 (12)	25	HCT 200 mg IV infusion daily for 4 or 8 days	45
Meduri et al. (2022)	584	96	68.8	PSI V: 119 (40) APACHE III: 54.3 ± 29.4	PSI V: 113 (40) APACHE III: 53.4 ± 28.7	297 (100)	287 (100)	47 (16)	50 (18)	NR	MP 40 mg IV bolus, than the full 20-day treatment course included 40 mg/day on days 1–7, 20 mg/day on days 8–14, 12 mg/day on days 15–17, and 4 mg/day on days 18–20	95.25
Wittermans et al. (2021)	156	67.4	67.5	PSI V: 9 (12)	PSI V: 13 (17)	NR	NR	3 (4)	5 (6)	20.45	DXM 6 mg PO daily for 4 days	24
Li et al. (2016)	58	NR	NR	NR	NR	29 (100)	29 (100)	6 (21)	6 (21)	NR	MP 80 mg IV infusion daily for 7 days	105
Blum et al. (2015)	386	62	73	PSI V: 54 (27)	PSI V: 54 (27)	NR	NR	15 (7)	13 (7)	16.15	prednisone 50 mg PO daily for 7 days	52.5
Torres et al. (2015)	120	61.4	65.3	PSI V: 22 (36)	PSI V: 19 (32)	43 (70)	47 (80)	6 (10)	9 (15)	25.87	MP 0.5 mg/kg IV infusion per 12 h for 5 days	65.6
Nafae et al. (2013)	70	56.2	49	NR	NR	NR	NR	4 (7)	6 (42)	9.23	HCT 200 mg IV bolus, then 10 mg/h IV infusion for 7 days	70.5
Fernández-Serrano et al. (2011)	43	66.7	61	NR	NR	NR	NR	1 (5)	1 (5)	NR	MP 200 mg IV bolus, then 20 mg/6 h IV infusion for 3 days, then 20 mg/12 h IV infusion for 3 days, then 20 mg/24 h IV for 3 days	116.3
Sabry and Omar (2011)	80	72.5	62.2	SOFA: 8.5 ± 1.5	SOFA: 8.2 ± 1.5	40 (100)	40 (100)	2 (5)	6 (15)	56.85	HCT 200 mg IV infusion, followed by 300 mg daily for 7 days	86.3
Snijders et al. (2010)	93	58.2	63.5	PSI V: 13 (27)	PSI V: 17 (38)	NR	NR	5 (10)	5 (11)	23.59	Prednisolone 40 mg IV infusion or PO, as appropriate, daily for 7 days	42
El-Ghamrawy et al. (2006)	34	61.8	61.8	NR	NR	17 (100)	17 (100)	3 (18)	6 (35)	NR	HCT IV 200 mg IV bolus, then 240 mg daily for 7 days	70.5
Confalonieri et al. (2005)	46	69.5	63.5	APACHE II: 17.2 ± 4.1	APACHE II: 18.2 ± 4.0	23 (100)	23 (100)	0 (0)	7 (30)	42	HCT IV 200 mg IV bolus, then 10 mg/h for 7 days	70.5
Marik et al. (1993)	30	NR	36.4	APACHE II: 11 ± 2	APACHE II: 14 ± 6	14 (100)	16 (100)	1 (7)	3 (19)	NR	HCT single dose 10 mg/kg IV infusion	26.25

CS: corticosteroids; PC: placebo or control; HCT: hydrocortisone; MP: methylprednisolone; DXM: dexamethasone; OD: once-daily; BD: twice-daily; IV: intravenous; PO; Per-Oral; mg: milligram; NR: not reported; ATS: american thoracic society, BTS: british thoracic society; PSI: pneumonia severity index; ICU: Intensive Care Unit.

### Risk-of-bias assessments

Risk-of-bias assessments are shown in Supplementary Figure S1. Eleven trials (Dequin et al., 2023; Meduri et al., 2022; Wittermans et al., 2021; Torres et al., 2015; Snijders et al., 2010; Sabry and Omar, 2011; Nafae et al., 2013; Fernández-Serrano et al., 2011; El-Ghamrawy and Esmat, 2006; Confalonieri et al., 2005; Blum et al., 2015) were assessed as low risk, while two (Marik et al., 1993; Li et al., 2016) were assessed as high risk.

### Different types of corticosteroids for 30-day mortality

**FIGURE 2:**
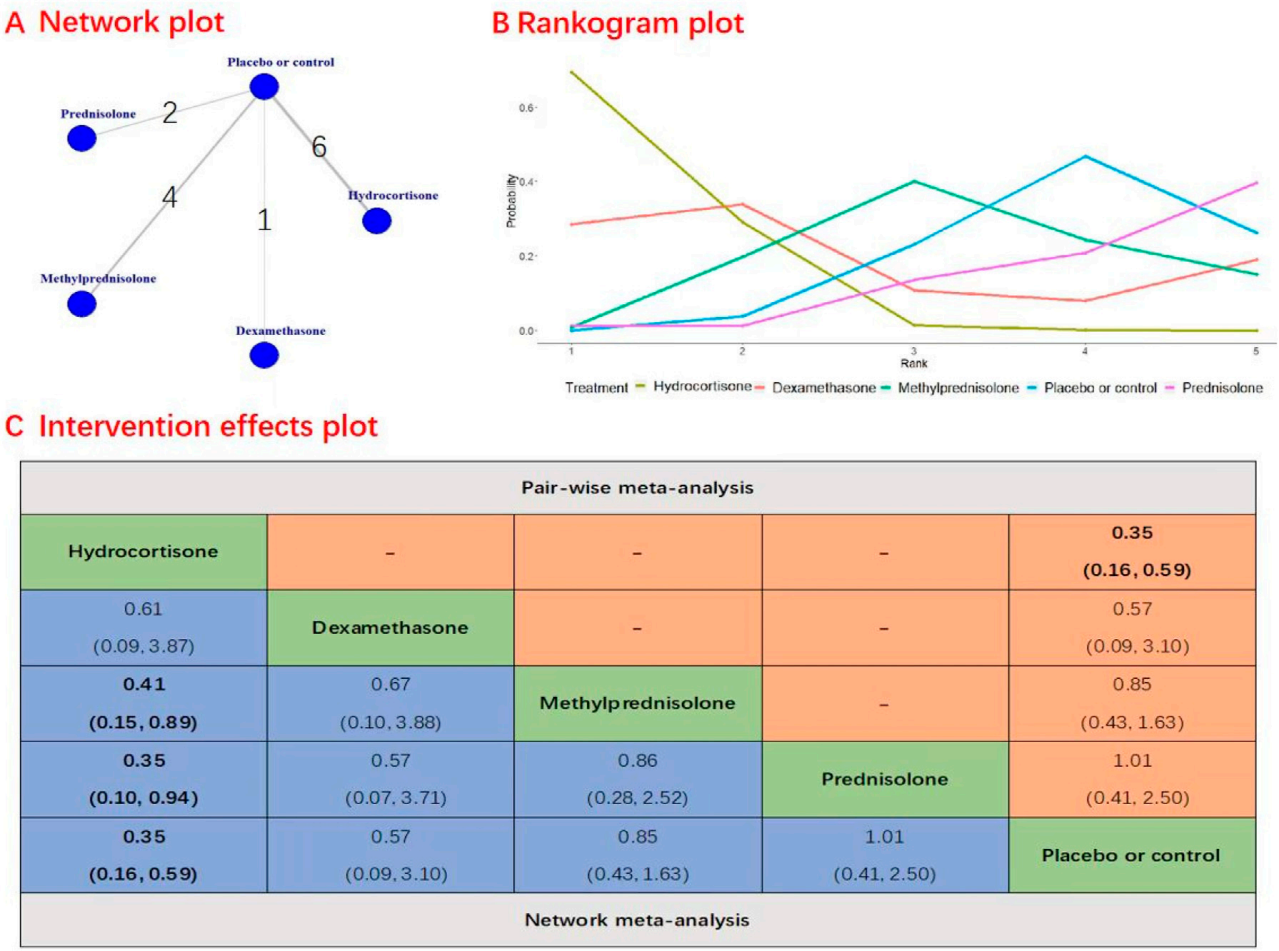
Network plot, rankogram plot and intervention effects plot for types of corticosteroid comparisons for 30-day mortality. **(A)** Network Plot illustrates the connections between various types of corticosteroids and the control group in network meta-analysis for 30-day mortality. Each node represents a treatment, with edges indicating direct comparisons. **(B)** Rankogram Plot displays the probability ranking of each corticosteroid treatment for reducing 30-day mortality. Each line represents a treatment and indicates the probability of achieving a specific rank. **(C)** Intervention Effects Plot summarizes the pairwise and network meta-analysis outcomes, presenting the odds ratios and CI for each corticosteroid type relative to others and to the control group in terms of 30-day mortality.

A total of 13 studies with 2,495 patients were included in the evaluation of the efficacy and safety of different types of corticosteroids on 30-day mortality. Figure 2 presents the results of Network plot, rankogram plot, and intervention effects plot (both pairwise meta-analyses and network meta-analysis) for types of corticosteroid comparisons for 30-day mortality. In direct comparison to placebo, the administration of hydrocortisone (RR, 0.35; 95% CI, 0.16–0.59; quality of evidence: moderate) significantly reduced the risk of 30-day mortality. There was no evidence to support the notion that prednisolone (RR, 1.01; 95% CI, 0.41–2.50), methylprednisolone (RR, 0.85; 95% CI, 0.43–1.63), or dexamethasone (RR, 0.57; 95% CI, 0.09–3.10) decreased 30-day mortality when compared to placebo. But there was evidence to support the notion that hydrocortisone is more effective than prednisone (RR, 0.35; 95% CI, 0.10–0.94; quality of evidence: moderate) and methylprednisolone (RR, 0.41; 95% CI, 0.15–0.89; quality of evidence: moderate) at reducing 30-day mortality. However, no significant difference was observed between hydrocortisone and dexamethasone in this regard (RR, 0.61; 95% CI, 0.09–3.87; quality of evidence: low). Notably, the rankogram plot indicated that hydrocortisone had the highest statistical probability of being the optimal choice for 30-day mortality (Figure 2). Hydrocortisone had the highest SUCRA value 0.70, followed by prednisone (SUCRA, 0.39), and methylprednisolone (SUCRA, 0.40).

The sensitivity analyses, which involved the exclusion of the trial by Marik et al. (1993) that used a single dose of corticosteroids (Supplementary Figure S2), the exclusion of studies with less severe disease conditions (Supplementary Figure S3), and the exclusion of studies that included patients with C-reactive protein levels below 15 mg/dL (Supplementary Figure S4) and were conducted by exclusively focusing on RCTs with a low risk of bias (Supplementary Figure S4), yielded similar results for 30-day mortality. These findings further enhanced the robustness and reliability of our results.

Our subgroup analysis indicates that higher cumulative doses of hydrocortisone are more effective than prednisolone, methylprednisolone, and placebo in reducing 30-day mortality in patients with SCAP. However, low cumulative doses of hydrocortisone showed no statistically significant difference compared to other types of corticosteroids. Therefore, we call for more RCTs in the future to ascertain the optimal dosage and duration of hydrocortisone treatment for SCAP (Supplementary Figure S6, The potential scale reduction factor value of 1.000 suggested a strong iterative effect, complete convergence, and stable model outputs. The S3 table presented the main findings of the GRADE assessment of certainty for the outcome. Accordingly, hydrocortisone yielded benefits on mortality across all baseline risk categories (low to moderate certainty evidence).

### Secondary outcome

Among the trials, 5 investigated the use of hydrocortisone and 1 explored methylprednisolone for the secondary outcome. Hydrocortisone was more effective than placebo (RR, 0.53; 95% CI, 0.30–0.83; Supplementary Figure S7) in reducing the need for mechanical ventilation. However, no significant distinction was observed between hydrocortisone and methylprednisolone (RR, 1.02; 95% CI, 0.25–4.31). For safety outcomes, no significant differences were observed between different types of corticosteroids regarding the safety outcomes, including gastrointestinal bleeding (Supplementary Figure S8) and healthcare-associated infections (Supplementary Figure S9).

## Discussion

This network meta-analysis found that hydrocortisone is more effective than prednisolone, methylprednisolone, and placebo in reducing 30-day mortality, these results must be interpreted with caution due to the study’s limitations. Further RCTs are necessary to substantiate these findings.

Previous meta-analyses (Wu et al., 2023; Wu et al., 2018) have relied on pairwise techniques to estimate the use of steroids in adult patients with SCAP and have suggested the effectiveness of steroids in treating this condition. However, our study distinguishes itself by employing a network meta-analysis approach, which is a novel method in this field. Additionally, it focuses specifically on the therapeutic effects of steroids. It is worth noting that corticosteroids, as a group, possess immune, metabolic, and fluid homeostatic features. However, each drug within this group exhibits different levels of activity in these areas. Our network meta-analysis provides a comprehensive evaluation of the treatment efficacy of different steroids. Results indicate a high probability of the efficacy of hydrocortisone treatments in terms of reducing mortality. Consistent with our findings, Wu (Wu et al., 2023) et al. conducted a meta-analysis to evaluate the effect of corticosteroids in patients with SCAP and performed a subgroup analysis, and conducted statistical significance tests based on different types of corticosteroids. The results also found that hydrocortisone may be a better choice compared to other alternatives. However, in our network meta-analysis, hydrocortisone did not show a significant reduction in mortality compared to dexamethasone (RR, 0.61; 95% CI, 0.09–3.87), although the mathematical statistical analysis did not reveal any significant differences, it is important to note that our analysis included only one trial focusing on dexamethasone, involving just 156 patients. This limitation suggests that the results should be interpreted with caution. This underscores the need for additional RCTs to thoroughly evaluate the efficacy of hydrocortisone vs dexamethasone in SCAP. The latest updated guidelines, such as the Society of Critical Care Medicine’s 2024 Focused Update (Chaudhuri et al., 2024) and the ERS/ESICM/ESCMID/ALAT guidelines (Estrada and Restrepo, 2024), recommend the use of corticosteroids in SCAP, but the quality of evidence supporting these recommendations is low, and they do not specify which corticosteroid is most effective for treating severe CAP. So our study not only enhances the existing body of evidence but also provides further support for the use of hydrocortisone in treating severe CAP, which may help to update these guidelines. As with any therapeutic intervention, it is crucial to consider individual patient factors, potential risks, and the need for careful monitoring during treatment.

Two trials were assessed as high-risk for bias (Marik et al., 1993; Li et al., 2016). These studies were included in the overall analysis, but we conducted sensitivity analyses excluding high-risk studies to ensure the robustness of our findings. The exclusion of these high-risk studies did not significantly alter the primary outcome of 30-day mortality, indicating that their inclusion did not have a major impact on the overall results.

Several potential explanations may exist for why hydrocortisone is more effective with SCAP. First, hydrocortisone, a short-acting and low-potency glucocorticoid, effectively moderates the immune response and reduces inflammation without significant immune system activation (Meduri et al., 2020). In comparison, prednisolone, methylprednisolone, and dexamethasone are more potent, longer-acting glucocorticoids that may cause extended immunosuppression, potentially impairing the body’s infection-fighting capabilities. Due to its shorter action duration and lower potency, hydrocortisone minimizes the risk of severe immunosuppression while still providing anti-inflammatory benefits. These properties are thought to contribute to its efficacy in decreasing mortality in SCAP. Second, Hydrocortisone acts as a glucocorticoid with notable mineralocorticoid properties (Hicks et al., 2012), influencing the regulation of both sodium and potassium. In contrast, synthetic glucocorticoids such as dexamethasone and methylprednisolone mainly demonstrate glucocorticoid activity with minimal impact on mineralocorticoid functions (Shibata, 2017). The dual functionality of hydrocortisone, encompassing both glucocorticoid and mineralocorticoid effects, potentially offers unique therapeutic advantages. This dual action is particularly valuable in the management of SCAP, where maintaining fluid balance and stable blood pressure is crucial under conditions of significant stress. Third, the intermediate half-life of hydrocortisone may allow for flexible dosing to achieve and maintain therapeutic levels with minimized risk of complications from prolonged high doses (Bonnin et al., 2021; Arafah, 2020).

The criteria for SCAP varied across the included studies, which introduced heterogeneity into our analysis. This variability may have influenced the findings, particularly in terms of mortality outcomes. To account for this, we applied a random-effects model that accommodates variability across studies. In addition, we performed sensitivity analyses by excluding studies with less severe disease conditions, those that used single-dose corticosteroids, and studies where patients had C-reactive protein levels below 15 mg/dL. These methods allowed us to better manage heterogeneity, ensuring the robustness and reliability of our conclusions. Further research is warranted to address this variability through more standardized definitions of SCAP. In our study, individual patient differences, such as age, gender, and underlying diseases, may significantly influence responses to glucocorticoid treatment. These differences not only affect the inflammatory response of patients but may also alter the effectiveness and safety of the treatment. Therefore, future studies should consider these factors and conduct subgroup analyses to better understand the responses of different patient populations to glucocorticoids. This understanding will help optimize treatment regimens, making them more personalized and effectively addressing the needs of various patients. In this study, we examined the efficacy of various types of corticosteroids in patients with SCAP and recognized that interactions between corticosteroids and other medications could significantly impact patient outcomes. Corticosteroids (such as hydrocortisone, prednisolone, and dexamethasone) enhance the effectiveness of antibiotics through immunomodulatory mechanisms; however, their immunosuppressive effects may, in certain circumstances, increase the risk of infections, thereby affecting the efficacy of antibiotics. The concurrent use of corticosteroids with anticoagulants (such as warfarin) may elevate the risk of bleeding, necessitating careful consideration and regular monitoring of patients' coagulation parameters by clinicians. Furthermore, the combination of corticosteroids with sedatives (such as benzodiazepines) may result in excessive sedation and respiratory depression. Therefore, clinicians should be attentive to the overall medication regimens of patients to ensure the safety of the treatment. Developed countries typically possess more advanced healthcare infrastructure and resources, allowing for broader and more standardized application of glucocorticoids in the treatment of SCAP. For example, clinical guidelines in these nations are often more explicit in recommending the use of glucocorticoids, and physicians demonstrate higher awareness and adherence to these protocols. Additionally, patients in developed countries generally have better access to high-quality medical services and consistent medication supplies, which can enhance treatment efficacy. In contrast, developing countries may face challenges such as limited healthcare resources, inadequate drug supply, and poor implementation of clinical guidelines. These obstacles can result in lower usage rates or inconsistent application of glucocorticoids in SCAP treatment. Furthermore, economic factors and healthcare costs play a significant role in influencing glucocorticoid use, as patients in developing regions may reduce their use of these medications due to financial constraints. These geographical disparities can lead to variations in patient responses to glucocorticoid therapy for SCAP across different regions, thereby affecting the generalizability and applicability of our study findings.

In this study, we place significant emphasis on ethical considerations, particularly the importance of informed consent. Clinicians have a crucial responsibility to ensure that patients are fully informed about the potential risks and benefits associated with glucocorticoid treatment before proceeding. This includes a comprehensive discussion of how glucocorticoids may impact their health, possible side effects, and the overall treatment strategy. It is essential that patients not only receive this information but also understand their treatment options. This dialogue fosters a trusting relationship between clinicians and patients, empowering individuals to make informed decisions about their care and treatment pathways. In our study, the potential for publication bias may significantly impact the results. Publication bias refers to the phenomenon where positive findings are more likely to be published, while negative or non-significant results may be overlooked. This bias can lead to an overestimation of the effectiveness of hydrocortisone in reducing 30-day mortality. Therefore, although our findings indicate that hydrocortisone has a substantial advantage in treating SCAP, we must interpret these results with caution and recognize that publication bias may influence the reliability of our conclusions. To mitigate this risk, future research should ensure that all relevant studies are appropriately published, regardless of the significance of their findings. This approach will help provide a more comprehensive and reliable evidence base. We thoroughly examined the impact of varying dosages and routes of administration on the use of corticosteroids in the treatment of SCAP. Research indicates that the choice of corticosteroid dosage directly affects both its efficacy and safety. Higher dosages may enhance anti-inflammatory effects in the short term; however, they also increase the risk of adverse effects, such as infections and metabolic disorders. Therefore, it is essential to balance the therapeutic benefits of dosage with its potential risks in clinical applications. Furthermore, the choice of administration route is equally critical. Intravenous administration is typically employed for severely ill patients to ensure rapid and effective drug absorption, while oral administration is more suitable for those with mild conditions. This distinction can lead to pharmacokinetic variations within the body, subsequently influencing treatment outcomes. Consequently, selecting the appropriate administration route not only improves efficacy but also minimizes side effects. We emphasize that the interplay between these factors significantly impacts the treatment outcomes for SCAP patients, necessitating that clinicians carefully consider them when formulating treatment plans. Therefore, future research should further investigate the effects of different dosages and administration routes on treatment outcomes to provide more precise, individualized therapy for SCAP patients. Patient adherence is crucial in the treatment of SCAP. Although we assessed adherence in the included studies and found satisfactory levels, we acknowledge the need to enhance patient adherence in practical treatment settings. Therefore, we recommend that future research adopt the following strategies: strengthen patient education, implement regular follow-ups, and develop personalized treatment plans to improve patient engagement and adherence to therapy.

Patients with SCAP typically present with varying degrees of basal inflammation, with C-reactive protein being a commonly used clinical marker to reflect this inflammation (Sproston and Ashworth, 2018). C-reactive protein, an acute-phase reactant, significantly increases during inflammatory responses. Higher C-reactive protein levels usually indicate more severe inflammation and infection, suggesting a more pronounced systemic inflammatory response. In SCAP, elevated C-reactive protein levels can identify patients experiencing a more intense inflammatory reaction, making them potentially more responsive to the anti-inflammatory effects of corticosteroids. To address C-reactive protein as a confounding factor, we conducted a sensitivity analysis excluding studies that included patients with C-reactive protein levels below 15 mg/dL. The results indicate that our findings remain robust even after excluding patients with C-reactive protein levels below 15 mg/dL. Future RCTs should focus on verifying the response of patients with different C-reactive protein levels to corticosteroid treatment and exploring the optimal C-reactive protein threshold to more accurately guide clinical decision-making. Additionally, integrating research on other inflammatory markers will provide a more comprehensive basis for patient assessment. These studies should include incorporating baseline C-reactive protein measurement in the study design to predict treatment outcomes, continuously monitoring C-reactive protein levels during treatment to evaluate progress and efficacy, conducting larger-scale RCTs to enhance the statistical power and generalizability of the results, and investigating the specific role and potential mechanisms of C-reactive protein levels in corticosteroid treatment. These improvements will elucidate the relationship between C-reactive protein levels and the efficacy of corticosteroid therapy, providing a scientific basis for clinical practice.

Future research should focus on several key areas. First, further investigations into dosage and treatment duration are essential. We recommend that upcoming RCTs concentrate on identifying the most effective corticosteroid treatment regimens, taking into account varying dosages and treatment lengths to tailor them to patients' severity and inflammatory responses. Second, assessing baseline inflammation is critical; future studies should explore the relationship between baseline inflammatory markers, particularly C-reactive protein levels, and the efficacy of corticosteroid therapy to optimize patient selection criteria and treatment protocols. Additionally, subsequent research should include direct comparisons of different glucocorticoids to establish their relative effectiveness and safety in treating SCAP. Lastly, given the potential effects of corticosteroids on blood glucose levels, future trials should closely monitor these levels to evaluate associated risks. By addressing these areas, the research should not only validate existing findings but also comprehensively assess safety and effectiveness to refine treatment guidelines. Several limitations need to be acknowledged in this study. Firstly, there is inconsistency in the definition of SCAP among the included studies as different researchers utilized various criteria, which may introduce potentially significant heterogeneity. For instance, Wittermans et al. relied on the Pneumonia Severity Index, Torres et al. considered patients admitted to the Intensive Care Unit or with a high Pneumonia Severity Index score, while Meduri et al. utilized the ATS/IDSA guidelines to define SCAP. Therefore, when applying the GRADE to assess the primary outcome, the certainty of outcomes had been rated down due to inconsistency. Secondly, the study lacks direct comparisons of treatment regimens, which may limit the robustness of the inference in the network meta-analysis, hinder the evaluation of consistency between direct and indirect evidence, and weaken the certainty of the conclusions. Therefore, caution is necessary when explaining this outcome due to the potential uncertainty behind the conclusions. Additionally, while hydrocortisone shows significant benefits, it is important to acknowledge the limited data available on other corticosteroids, such as dexamethasone. Our analysis only included one trial focusing on dexamethasone, involving a small sample size. This limitation suggests that further direct comparison trials are needed to thoroughly evaluate the efficacy of dexamethasone and other corticosteroids in SCAP. Thirdly, due to the lack of original RCTs reporting on long-term outcomes and the effects of corticosteroids on blood glucose levels, we are unable to provide data on long-term efficacy. This limitation impacts our comprehensive assessment of treatment effectiveness. Additionally, we acknowledged the potential for corticosteroids to cause fluctuations in blood glucose levels, which could lead to adverse events. However, the majority of the included studies did not provide data on blood glucose levels, thereby limiting our ability to assess this effect. We hope that future RCTs will investigate this aspect more thoroughly. Fourthly, our systematic review and network meta-analysis are limited by the potential confounding effects of corticosteroid dosage and duration. Variability in these parameters across the included studies could affect the generalizability of our conclusions. Therefore, we advocate for future RCTs to determine the optimal dosage and duration of corticosteroid treatment for SCAP. Fifthly, the studies included exhibit variations in basal inflammation, as measured by C-reactive protein levels. Although we conducted sensitivity analyses that excluded studies with C-reactive protein levels below 15 mg/dL, our results remained robust. However, basal inflammation (such as C-reactive protein level) is a crucial confounding factor that cannot be ignored. This limits our understanding of the efficacy of corticosteroids, such as hydrocortisone, in treating SCAP across different inflammatory contexts. Sixthly, while our sensitivity analysis primarily focused on C-reactive protein levels, other inflammatory markers, such as interleukins (e.g., IL-6, IL-10), may also play a significant role in the inflammatory response in CAP. IL-6 has been closely linked to disease severity and is a key marker in predicting the response to antimicrobial therapy (Rosolowski et al., 2020), while IL-10 plays a crucial role in modulating inflammation (Kühnapfel et al., 2022). Future studies should further investigate the regulatory effects of these markers on corticosteroid treatment, which could provide valuable insights for treatment decisions and disease management. Seventhly, this study has not sufficiently considered the impact of individual differences on responses to glucocorticoid treatment. Although we conducted sensitivity analyses, factors such as patient age, gender, and underlying diseases may lead to heterogeneity in the results. The lack of an in-depth exploration of these factors may limit our understanding of the generalizability of the findings. Therefore, we recommend that future RCTs take these individual differences into account in their designs to assess the efficacy and safety of glucocorticoid treatment comprehensively. Eighthly, the relatively small number of included studies and patients may affect the stability and generalizability of the results despite sensitivity analyses. Future research should involve larger, more diverse samples and standardized methodologies to validate and expand upon these results. Ninthly, geographical differences in the use of glucocorticoids for treating SCAP between developed and developing countries—due to variations in medical infrastructure, resources, and economic factors—may limit the applicability of our findings. Future research should involve larger, more diverse samples and standardized methodologies to validate and expand upon these results. Tenthly, this study was unable to provide a detailed analysis of the cost of glucocorticoids due to the lack of relevant data in the original RCTs. This is an important limitation of our study. Future research should include an assessment of economic benefits to more comprehensively compare the clinical outcomes and costs of different glucocorticoids, providing a basis for developing more cost-effective treatment strategies. Eleventhly, most of the studies included in this network meta-analysis did not evaluate patients' quality of life, which is a crucial assessment metric. Consequently, we are unable to fully determine the overall impact of corticosteroid therapy on patients with SCAP. Additionally, this study primarily focused on clinical outcomes, such as mortality, and lacked patient-reported quality of life data. Future RCTs should not only prioritize clinical endpoints but also incorporate quality of life assessments to provide a more comprehensive evaluation of corticosteroid treatment effects. Integrating quality of life data will aid in optimizing treatment strategies, achieving the dual goals of increasing survival rates and enhancing patients' quality of life. Future RCTs should focus on verifying the response of patients with different C-reactive protein levels to corticosteroid treatment and exploring the optimal C-reactive protein threshold to more accurately guide clinical decision-making. Finally, we must acknowledge that our network meta-analysis includes trials with significant heterogeneity, particularly concerning the choice of corticosteroids, daily and cumulative doses of corticosteroids, duration of treatment, time to treatment, and severity of the patient’s condition. Therefore, more RCTs are needed to validate our research results.

## Conclusion

While hydrocortisone demonstrated a higher probability of efficacy in reducing 30-day mortality compared to prednisolone, methylprednisolone, and placebo, these results must be interpreted with caution due to the study’s limitations. Further RCTs are necessary to substantiate these findings.

## Data Availability

The original contributions presented in the study are included in the article/supplementary material, further inquiries can be directed to the corresponding author.
