# Supplementary material for: Association between various types of corticosteroids and mortality for severe community-acquired pneumonia in adults: a systematic review and network meta-analysis
Source: Front Pharmacol. 2024 Nov 26;15:1479804. doi: 10.3389/fphar.2024.1479804 (PMC11628252; doi:10.3389/fphar.2024.1479804)

**Association between Various Types of Corticosteroids and Mortality for Severe Community-Acquired Pneumonia in Adults: A Systematic Review and Network Meta-Analysis**

**Authors:**

Peng Wang MD^1,2*^, Jun Wan MD^3*^, Qiaoyu You MD^1^, Yuxin Zheng MD^1^, Wenhao Xu MD^1^, Jialing He MD^2^, Liyuan Peng MD^5^, CUYUBAMBA DOMINGUEZ, JORGE LUIS MD^9^, Yuning Feng MD^1^, Ping Xu MD^8^, Xinrong Li MD^10^, Weelic Chong MD^6^, Yang Hai MD^7^, Lu Jia MD^4^, Fang Fang MD^2^, Yu Zhang MD^1^

**Affiliations**

1. Center for Evidence-based Medicine, Affiliated Hospital of Chengdu University, Chengdu, Sichuan, China
2. Department of Neurosurgery, West China Hospital, Sichuan University, Chengdu, Sichuan, China
3. Clinical Medical College & Affiliated Hospital of Chengdu University, Chengdu University
4. Department of Surgical Intensive care medicine, Shanxi Provincial People’s Hospital, Taiyuan, Shanxi, China.
5. Department of critical care medicine, Affiliated Hospital of Chengdu University, Chengdu, Sichuan, China
6. Sichuan University Library, Sichuan University, Chengdu, Sichuan, China
7. Sidney Kimmel Medical College, Thomas Jefferson University, Philadelphia, PA
8. Sichuan University Library, Chengdu, Sichuan, China.
9. Institutes for Systems Genetics, West China Hospital, Sichuan University, Chengdu, China.
10. Tangshan Research Institute, Southwest Jiaotong University, Tangshan, 063002, Hebei, China

Correspondence to: Yu Zhang, Center for Evidence-based Medicine, Affiliated Hospital of Chengdu University, No.82, North Section 2, 2nd Ring Road, Jinniu District, Chengdu, Sichuan 610081, China. Email: [zhangyu1057@cdu.edu.cn](mailto:zhangyu1057@cdu.edu.cn)

**Supporting information**

**S1 Table. Search strategy**

**S2 Table. Results and certainty assessments for the primary outcome comparing various types of corticosteroids for severe community-acquired pneumonia in adults.**

**S3 Table. Results and certainty assessments for the secondary outcome comparing various types of corticosteroids for severe community-acquired pneumonia in adults.**

**S1 Fig. Risk of bias summary**

**S2 Fig. Sensitivity analyses for excluding the trial by Marik et al. (using a single dose of corticosteroids)**

**S3 Fig. Sensitivity analyses for excluding studies with less severe disease conditions**

**corticosteroids)**

**S4 Fig. Sensitivity analyses for excluding study that included patients with C-reactive protein levels below 15 mg/dL**

**S5 Fig. Sensitivity analyses for exclusively focusing on randomized controlled trials with a low risk of bias**

**S6 Fig. Subgroup analysis based on the cumulative dexamethasone doses: lower cumulative doses of corticosteroids and higher cumulative doses of corticosteroids**

**S7 Fig. Network plot, forest plot and league plot of the network estimates for types of corticosteroid comparisons for the risk of requirement of mechanical ventilation.**

**S8 Fig. Network plot, forest plot and league plot of the network estimates for types of corticosteroid comparisons for gastrointestinal bleeding.**

**S9 Fig. Network plot, forest plot and league plot of the network estimates for types of corticosteroid comparisons for healthcare-associated infection.**

**Supporting information**

**S1 Table. Search strategy**

| **MEDLINE(R)** | | |
| --- | --- | --- |
|  | exp Adrenal Cortex Hormones/ | 422744 |
|  | exp STEROIDS/ | 917407 |
|  | (Adrenal Cortex Hormone* or adrenocortical hormone* or adrenocorticosteroid* or Corticosteroid* or Corticoid* or steroid* or glucocort* or cortisone* or hydrocortisone* or Cortisol or Epicortisol or Cortifair or Cortril or hydroxyhydrocortisone or oxohydrocortisone or tetrahydrocortisol or dexamethason* or baycuten or dexatopic or sofradex or Methylfluorpreordnisolone or Hexadecadrol or Decameth or Decaspray or Dexasone or Dexpak or Maxidex or Millicorten or Oradexon or Decaject or Decaject or Hexadrol or methylprednisolon* or (methyl adj3 prednisolone) or Metipred or Urbason or Medrol or Betamethasone or Flubenisolone or Betadexamethasone or Celestona or Cellestoderm or Celeston or Celestone or prednison* or prednisolon* or hydroxyprednisolone or desonide or Predate or Predonine or Di-Adreson-F or DiAdresonF or triamcinolon*).mp. | 799066 |
|  | or/1-3 | 1352273 |
|  | exp Pneumonia/ | 319369 |
|  | ((lung or pulmonary or lower respiratory) adj3 (inflammat* or infect*)).mp. | 65190 |
|  | (pneumoni* or bronchopneumoni* or pleuropneumoni*).mp. | 310316 |
|  | Community-Acquired Infections/ | 15906 |
|  | community acquired.mp. | 27239 |
|  | (5 or 6 or 7) and (8 or 9) | 17136 |
|  | ((communit* adj5 (pneumoni* or bronchopneumoni* or pleuropneumoni*)) or CAP).mp. | 58716 |
|  | 4 and (10 or 11) | 2095 |
|  | randomized controlled trial.pt. | 590910 |
|  | controlled clinical trial.pt. | 95268 |
|  | randomized.ab. | 599831 |
|  | placebo.ab. | 237464 |
|  | drug therapy.fs. | 2582224 |
|  | randomly.ab. | 406249 |
|  | trial.ab. | 644206 |
|  | groups.ab. | 2503884 |
|  | or/13-20 | 5632468 |
|  | 12 and 21 | 963 |
| **EMBASE** | | |
|  | exp corticosteroid/ | 1093886 |
|  | (Adrenal Cortex Hormone* or adrenocortical hormone* or adrenocorticosteroid* or Corticosteroid* or Corticoid* or steroid* or glucocort* or cortisone* or hydrocortisone* or Cortisol or Epicortisol or Cortifair or Cortril or hydroxyhydrocortisone or oxohydrocortisone or tetrahydrocortisol or dexamethason* or baycuten or dexatopic or sofradex or Methylfluorpreordnisolone or Hexadecadrol or Decameth or Decaspray or Dexasone or Dexpak or Maxidex or Millicorten or Oradexon or Decaject or Decaject or Hexadrol or methylprednisolon* or (methyl adj3 prednisolone) or Metipred or Urbason or Medrol or Betamethasone or Flubenisolone or Betadexamethasone or Celestona or Cellestoderm or Celeston or Celestone or prednison* or prednisolon* or hydroxyprednisolone or desonide or Predate or Predonine or Di-Adreson-F or DiAdresonF or triamcinolon*).mp. | 1447924 |
|  | 1 or 2 | 1523390 |
|  | community acquired pneumonia/ | 19454 |
|  | ((communit* adj5 (pneumoni* or bronchopneumoni* or pleuropneumoni*)) or CAP).mp. | 92023 |
|  | exp pneumonia/ | 387003 |
|  | ((lung or pulmonary or lower respiratory) adj3 (inflammat* or infect*)).mp. | 119719 |
|  | (pneumoni* or bronchopneumoni* or pleuropneumoni*).mp. | 526895 |
|  | community acquired infection/ | 4384 |
|  | community acquired.mp. | 41210 |
|  | (6 or 7 or 8) and (9 or 10) | 30613 |
|  | 4 or 5 or 11 | 96297 |
|  | 3 and 12 | 5129 |
|  | randomized controlled trial/ | 779167 |
|  | crossover procedure/ | 74839 |
|  | double blind procedure/ | 209257 |
|  | single blind procedure/ | 51405 |
|  | (random* or factorial* or crossover* or placebo* or assign* or allocat* or volunteer* or (doubl* adj5 blind*) or (singl* adj5 blind*)).mp. | 3180189 |
|  | or/14-18 | 3180189 |
|  | 13 and 19 | 723 |
|  | exp animal/ | 30493787 |
|  | human/ | 25190998 |
|  | 21 not 22 | 5302789 |
|  | 20 not 23 | 703 |
| COCHRANE CENTRAL | | |
|  | exp Adrenal Cortex Hormones/ | 31923 |
|  | exp STEROIDS/ | 67483 |
|  | (Adrenal Cortex Hormone* or adrenocortical hormone* or adrenocorticosteroid* or Corticosteroid* or Corticoid* or steroid* or glucocort* or cortisone* or hydrocortisone* or Cortisol or Epicortisol or Cortifair or Cortril or hydroxyhydrocortisone or oxohydrocortisone or tetrahydrocortisol or dexamethason* or baycuten or dexatopic or sofradex or Methylfluorpreordnisolone or Hexadecadrol or Decameth or Decaspray or Dexasone or Dexpak or Maxidex or Millicorten or Oradexon or Decaject or Decaject or Hexadrol or methylprednisolon* or (methyl adj3 prednisolone) or Metipred or Urbason or Medrol or Betamethasone or Flubenisolone or Betadexamethasone or Celestona or Cellestoderm or Celeston or Celestone or prednison* or prednisolon* or hydroxyprednisolone or desonide or Predate or Predonine or Di-Adreson-F or DiAdresonF or triamcinolon*).mp. | 99010 |
|  | or/1-3 | 136930 |
|  | exp Pneumonia/ | 9427 |
|  | ((lung or pulmonary or lower respiratory) adj3 (inflammat* or infect*)).mp. | 6427 |
|  | (pneumoni* or bronchopneumoni* or pleuropneumoni*).mp. | 22711 |
|  | Community-Acquired Infections/ | 655 |
|  | community acquired.mp. | 2217 |
|  | (5 or 6 or 7) and (8 or 9) | 1867 |
|  | ((communit* adj5 (pneumoni* or bronchopneumoni* or pleuropneumoni*)) or CAP).mp. | 5527 |
|  | 4 and (10 or 11) | 402 |

**S2 Table. Results and certainty assessments for the primary outcome comparing various types of corticosteroids for severe community-acquired pneumonia in**

| **Comparison** | Direct estimate | Network  estimate | Absolute Difference^*^  (95% CI) | GRADE |
| --- | --- | --- | --- | --- |
| Hydrocortisone vs. Placebo or control | 0.35(0.16,0.59) | 0.35(0.16,0.59) | -88(-56, -114) | Moderate^&^ |
| Hydrocortisone vs. Methylprednisolone | - | 0.41(0.15,0.89) | -87(-16, -125) | Moderate^&^ |
| Hydrocortisone vs. Prednisolone | - | 0.35(0.10,0.94) | -52(-5, -72) | Moderate^&^ |
| Hydrocortisone vs. Dexamethasone | - | 0.61(0.09,3.87) | -15(-35,112) | Low^#^ |
| Dexamethasone vs. Placebo or control | 0.57(0.09,3.10) | 0.57(0.09,3.10) | -58(-123,285) | Low ^#^ |
| Dexamethasone vs. Methylprednisolone | - | 0.67(0.10,3.88) | -48(-132,422) | Low ^#^ |
| Dexamethasone vs. Prednisolone | - | 0.57(0.07,3.71) | -34(-74,217) | Low ^#^ |
| Methylprednisolone vs. Prednisolone | - | 0.86(0.28,2.52) | -11(-58,122) | Low ^#^ |
| Methylprednisolone vs. Placebo or control | 0.85(0.43,1.63) | 0.85(0.43,1.63) | -20(-77,85) | Low ^#^ |
| Prednisolone vs. Placebo or control | 1.01(0.41,2.50) | 1.01(0.41,2.50) | 1(-80,203) | Low^#^ |

**adults.**

*****Absolute Effect Estimates, No. Of Patients Per 1000

# Imprecisions

& Inconsistency

**S3 Table. Results and certainty assessments for the secondary outcome comparing various types of corticosteroids for severe community-acquired pneumonia in adults.**

| **Comparison** | Estimates from network meta-analysis, Risk ratio (95% Cl) | | |
| --- | --- | --- | --- |
|  | requirement of mechanical ventilation | gastrointestinal bleeding | healthcare-associated infection |
| Hydrocortisone vs. Methylprednisolone | 1.02 (0.25, 4.31) | 1.24 (0.10, 17.09) | 0.46 (0.01, 6.08) |
| Hydrocortisone  vs. Placebo or control | 0.53 (0.30, 0.83) | 1.20 (0.47, 3.80) | 0.63 (0.064, 3.40) |
| Methylprednisolone vs. Placebo or control | 0.52 (0.13, 1.90) | 1.00 (0.096, 11.00) | 1.30 (0.16, 29.00) |

**S1 Fig. Risk of bias summary**

**
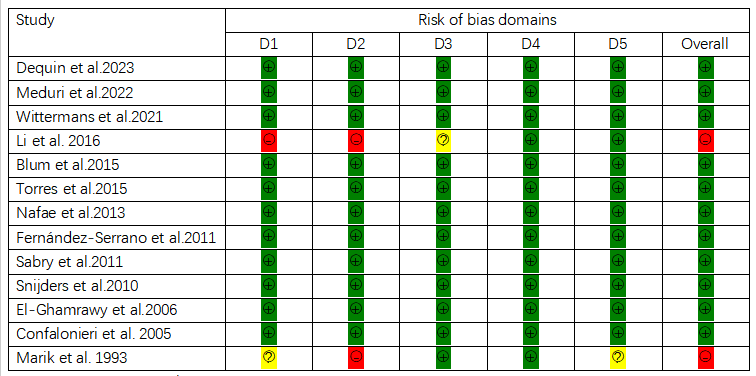
**

Domains:

D1: Bias arising from the randomization process.

D2: Bias due to deviations from intended intervention.

D3: Bias due to missing outcome data.

D4: Bias in measurement of the outcome.

D5: Bias in selection of the reported result.

Judgement:


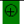
: Low risk of bias


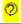
: some concerns risk of bias


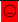
: High risk of bias

**S2 Fig. Sensitivity analyses for excluding the trial by Marik et al. (using a single dose of corticosteroids)** **
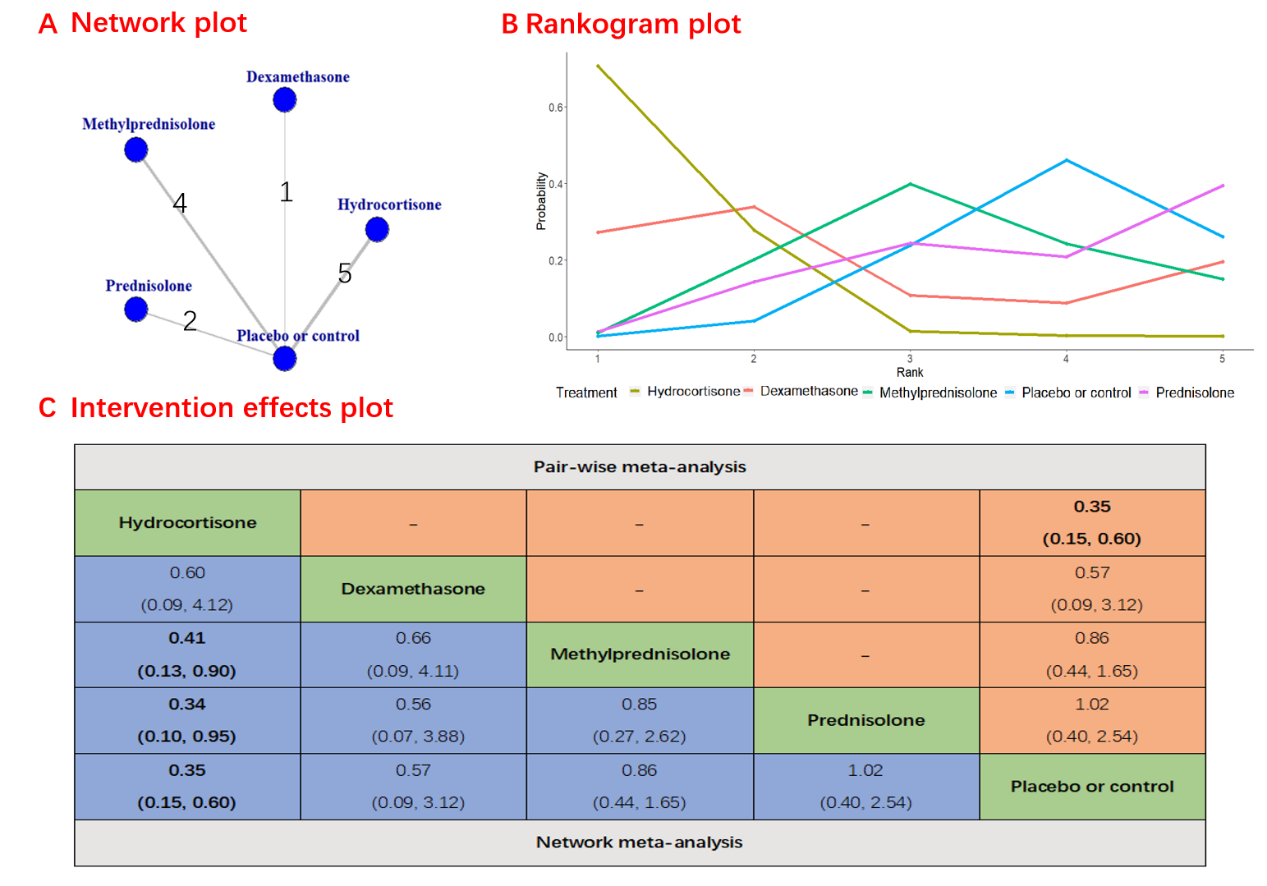
**

**S3 Fig. Sensitivity analyses for excluding studies with less severe disease conditions**

**
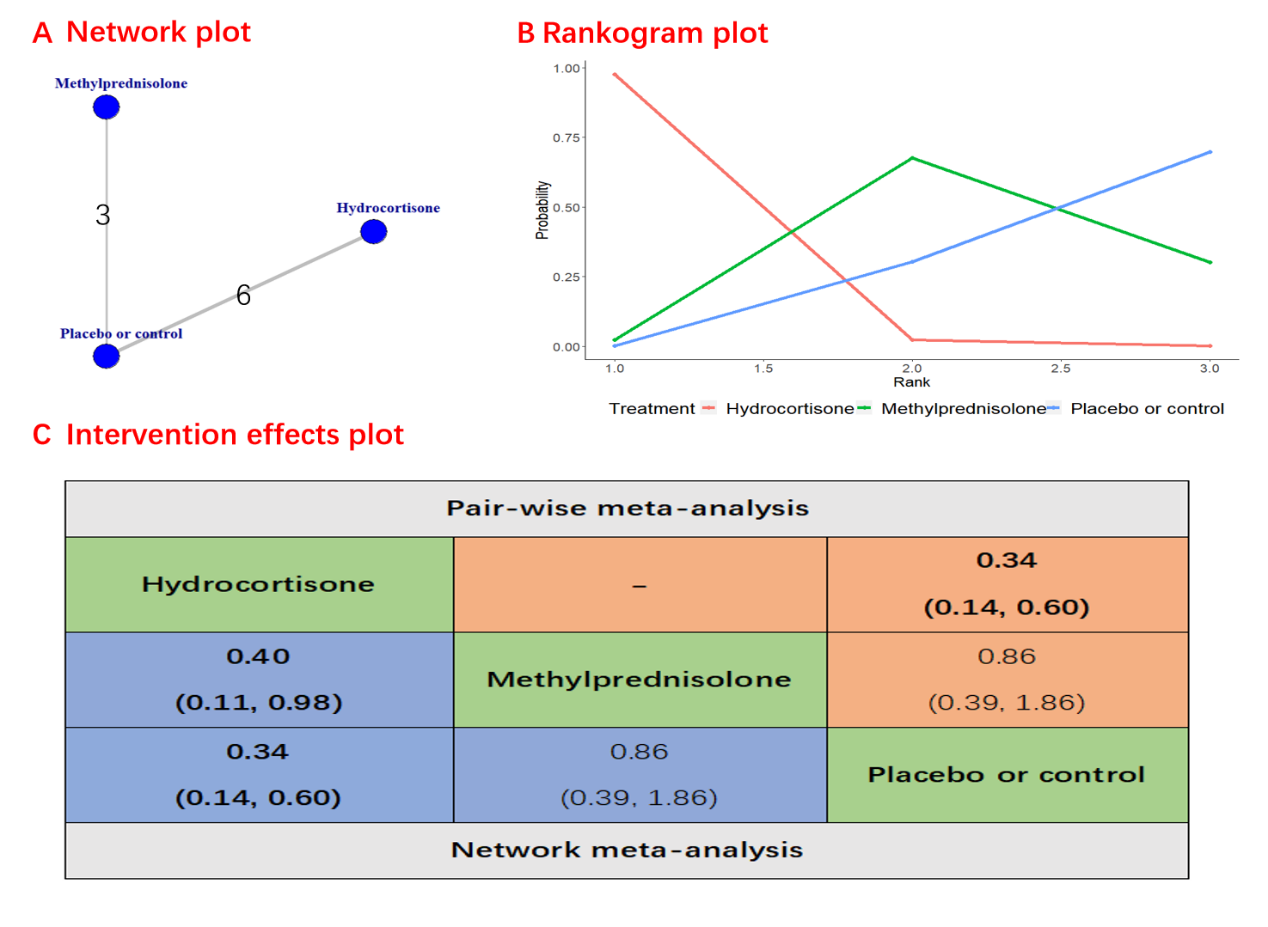
**

**S4 Fig. Sensitivity analyses for excluding study that included patients with C-reactive protein levels below 15 mg/dL**
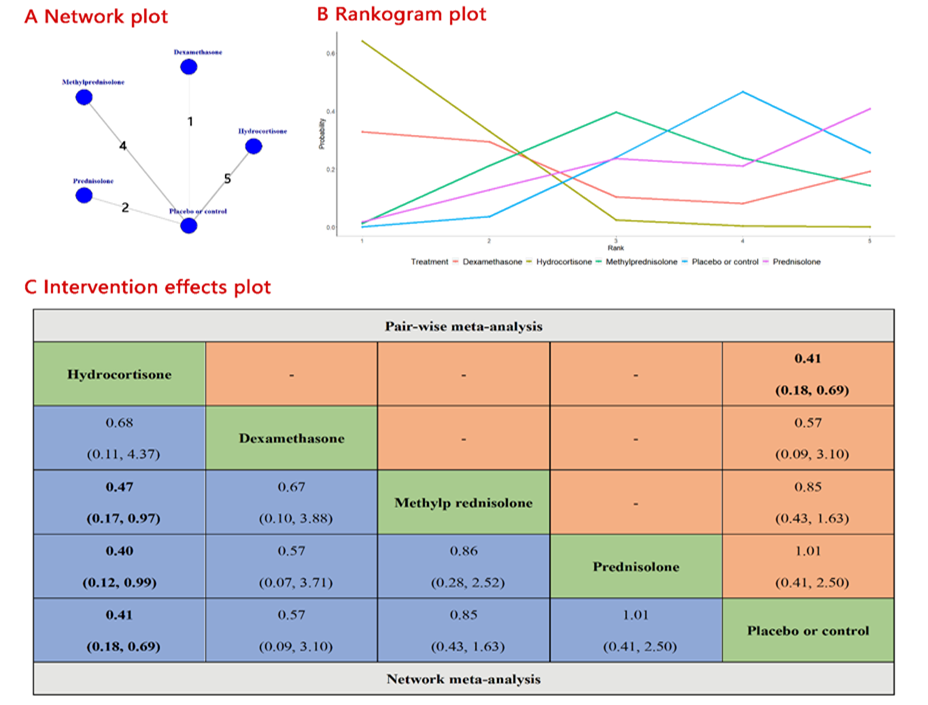


**S5 Fig. Sensitivity analyses for exclusively focusing on randomized controlled trials with a low risk of bias**


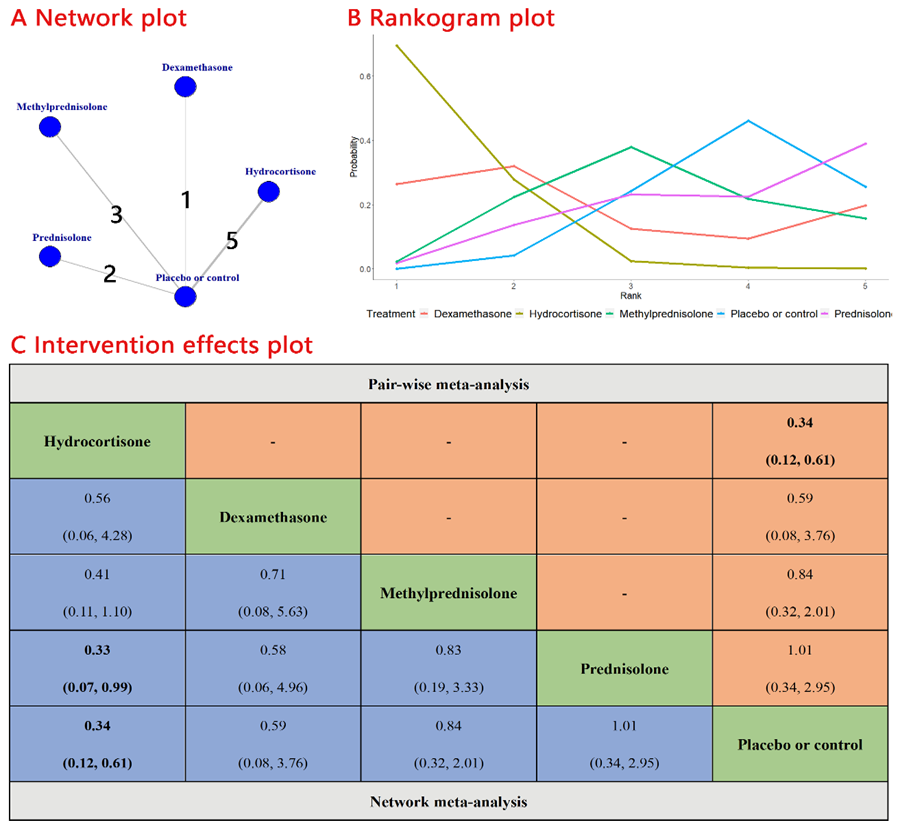
 **S6 Fig. Subgroup analysis based on the cumulative dexamethasone doses: lower cumulative doses of corticosteroids and higher cumulative doses of corticosteroids**
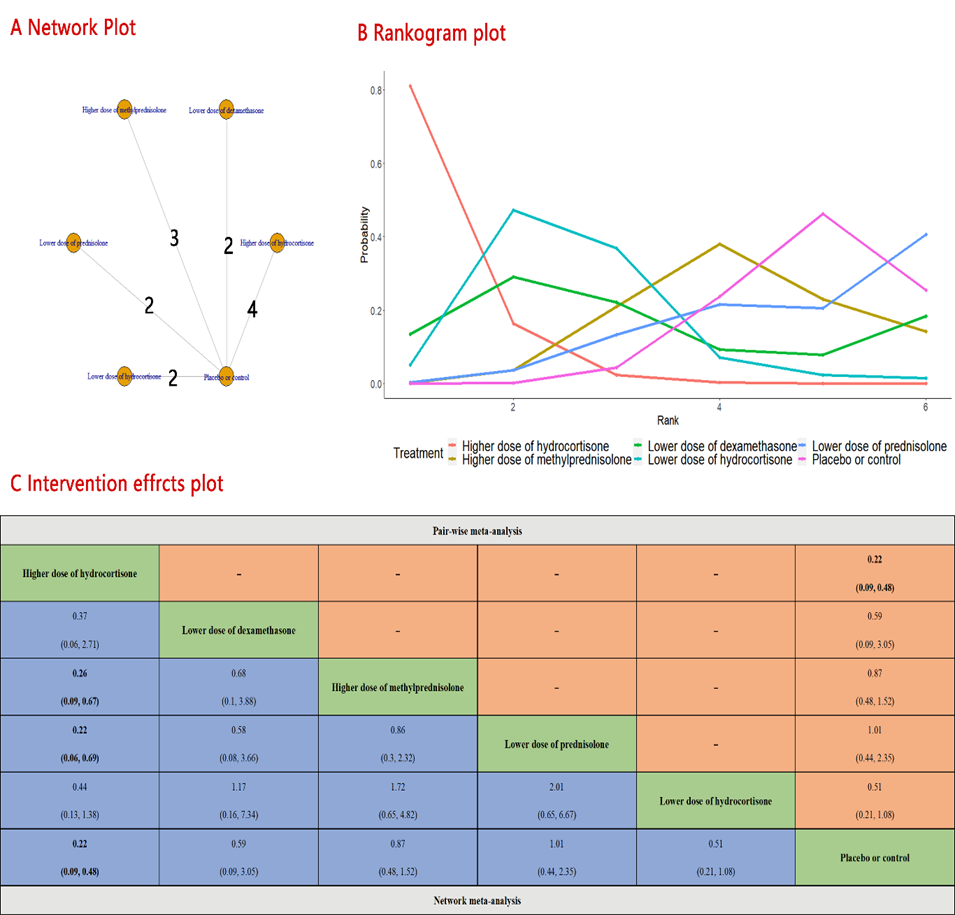


**S7 Fig. Network** **plot, forest plot and league plot of the network estimates for types of corticosteroid comparisons for the risk of requirement of mechanical ventilation.**
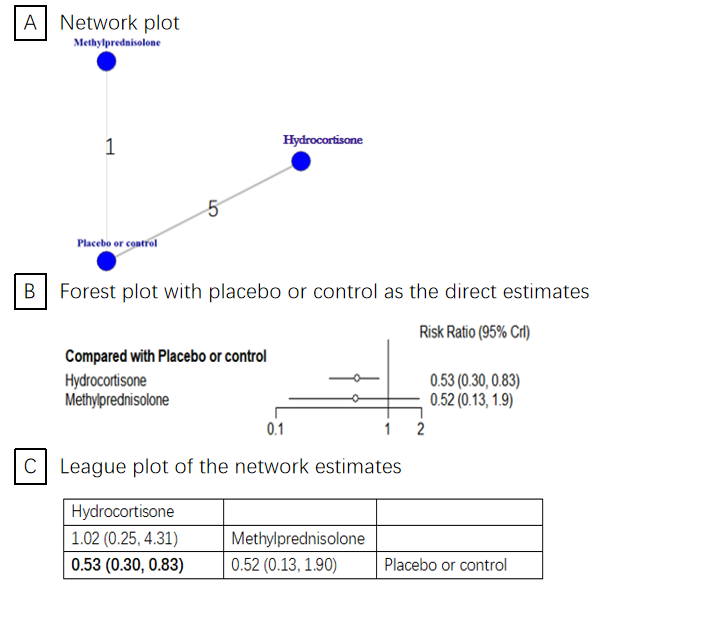


**S8 Fig. Network plot, forest plot and league plot of the network estimates for types of corticosteroid comparisons for gastrointestinal bleeding.**
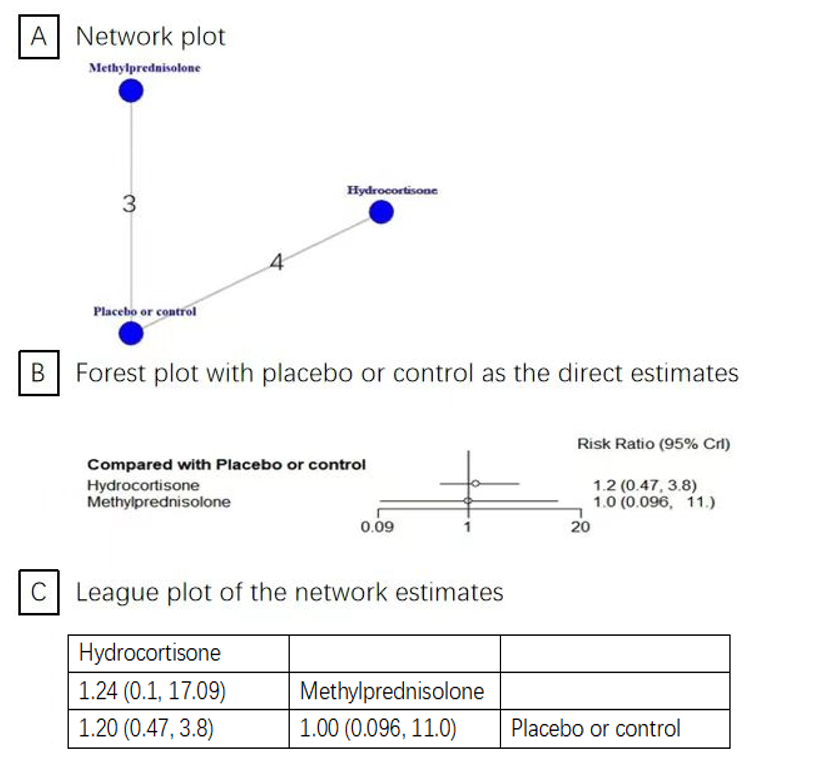


**S9 Fig. Network plot, forest plot and league plot of the network estimates for types of corticosteroid comparisons for healthcare-associated infection.**
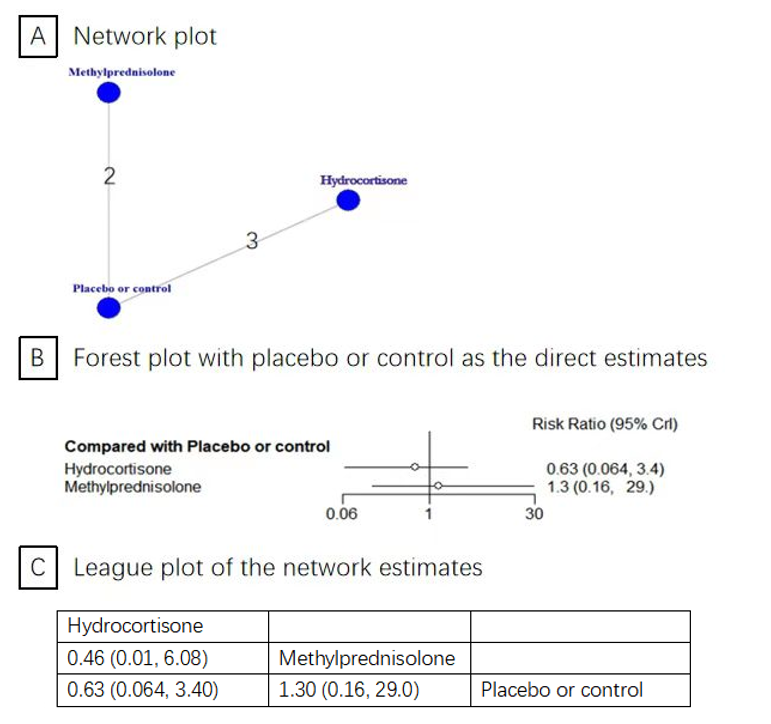

Supplement: Supplementary file 1 [file DataSheet1.docx]
